# Prolonged hypokalemia long after causative factor elimination in pseudo-Bartter/Gitelman syndrome

**DOI:** 10.1007/s10157-025-02734-4

**Published:** 2025-07-25

**Authors:** Atsushi Kondo, Tomoko Horinouchi, Yuta Inoki, Yuta Ichikawa, Yu Tanaka, Hideaki Kitakado, Chika Ueda, Nana Sakakibara, China Nagano, Kandai Nozu

**Affiliations:** Department of Pediatrics, Kobe University Graduate School of Medicine, 7-5-1, Kusunoki-cho, Chuo-ku, Kobe, Hyogo 650-0017 Japan

**Keywords:** Pseudo-Bartter/Gitelman syndrome, Hypokalemia, Renin–angiotensin system, Chronic kidney disease

## Abstract

**Background:**

Pseudo-Bartter/Gitelman syndrome (PBS/PGS) is caused by medication and lifestyle factors, leading to hypokalemia and potentially impairing kidney function. Treatment primarily involves eliminating the underlying causes, which typically results in rapid improvement. However, PBS/PGS findings may persist long after the removal of causative factors, and its pathogenesis remains unclear.

**Methods:**

This study focused on 49 cases diagnosed with PBS/PGS. All cases presented with hypokalemia, attributed to apparent causes, and comprehensive genetic testing detected no pathogenic variants associated with hereditary kidney diseases. They were categorized into two groups: the current group (*n* = 39), where causative factors persisted, and the past group (*n* = 10), where more than 1 year had elapsed since the elimination of the causative factors at the time of examination. A retrospective comparative analysis was conducted between these groups.

**Results:**

All patients were female, except for two in the current group. The median time since the elimination of causes in the past group was 7.5 years. Hypokalemia and kidney dysfunction were observed in both groups without statistically significant differences. Both groups exhibited overactivation of renin-angiotensin systems.

**Conclusion:**

This study is the first to reveal the possibility of persistent PBS/PGS findings even after the removal of causative factors. While swift removal of the cause of PBS/PGS is crucial, long-term post-removal monitoring is essential to improve renal prognosis.

**Supplementary Information:**

The online version contains supplementary material available at 10.1007/s10157-025-02734-4.

## Introduction

Gitelman syndrome (GS) is an inherited salt-losing tubulopathy (SLT), which is caused by loss-of-function mutations in the *SLC12A3* gene on chromosome 16q13 [1]. This gene encodes for a thiazide-sensitive sodium chloride co-transporter located in the distal convoluted tubule of the kidney. When its function is lost or weakened, massive sodium loss in urine occurs, leading to a compensatory loss of potassium and other substances. This results in hypokalemia, metabolic alkalosis, hypomagnesemia, and hypocalciuria. In general, GS has a milder clinical course compared to Bartter syndrome (BS). The onset of GS is common later in school age or adulthood, whereas BS is one of the SLTs that mostly occurs in the fetal to infantile period [1] [2]. The symptoms of GS are nonspecific and include the following: salt cravings, muscle spasms, muscle weakness, and fatigability. Renal prognosis is usually considered to be good. However, in a large quality of life (QOL) survey in patients with GS, it was revealed that patients suffering from these symptoms had a significant reduction in their QOL [3].

Alternatively, there are some cases in which patients develop symptoms similar to those of GS, despite the absence of any pathogenic variants in SLT-related genes, which is known as pseudo-Bartter/Gitelman syndrome (PBS/PGS) [2,4]. The causes of this condition may vary, including some medications that can cause hypokalemia such as diuretics or laxatives, severe hyperemesis gravidarum, alcoholism, anorexia nervosa, excessive dieting, and some other diseases like cystic fibrosis. Among these, long-term regular use of laxatives is reported to be the most common cause [4]. An important clinical feature of PBS/PGS is that these patients are known to be more prone to renal dysfunction compared to patients with GS ^[[4]]^. In addition, there are some reports of end-stage kidney disease (ESKD) in patients with PGS due to anorexia nervosa or persistent laxative abuse [5–7]. As no fundamental treatment for PBS/PGS has been established, the main treatments are the elimination of the underlying causes and potassium supplementation. In many cases, such as pregnancy-induced PBS/PGS, electrolyte abnormalities improve immediately once the apparent cause is resolved [8]. However, in some cases, recovery of the electrolyte abnormalities may take a long time after the elimination of causative factors. We encountered several cases in which hypokalemia persisted for years after the cause was resolved, and renal function deteriorated. Although such cases are clinically significant, there have been no reports investigating cases with prolonged hypokalemia and progressive renal dysfunction long after the elimination of the underlying causes of PBS/PGS.

In this study, we examined cases of patients diagnosed with PBS/PGS who underwent comprehensive genetic analysis for suspected SLTs. We compared the clinical manifestations of patients with clear and persistent causes of PBS/PGS at diagnosis with those who only had causes of PBS/PGS in the past.

## Methods

### Patient population and data collection

A total of 692 patients suspected of having SLT underwent comprehensive genetic testing at our institution between April 2006 and April 2023. This testing involved targeted exome sequencing using next-generation sequencing (NGS) which included screening for copy number variations (CNVs), multiplex ligation‐dependent probe amplification (MLPA), array comparative genomic hybridization (array CGH), and conventional direct sequencing using the Sanger method.

Patient clinical profiles were extracted from the genetic test request forms written by attending physicians and reviewed retrospectively. All patients presented with hypokalemia, which was defined as a serum potassium level of < 3.5 mEq/L, and based on their clinical findings and a thorough examination of potential causes, the attending physician ruled out other associated conditions such as primary aldosteronism, renal tubular acidosis, neoplastic disease, or adrenal hyperplasia.

### Diagnostic criteria for PBS/PGS and study design

In this study, we focused on 157 cases in which no pathogenic variant or CNVs was detected despite comprehensive genetic testing including targeted NGS for 136 genes related to inherited kidney diseases (Supplementary Table 1) described later. Cases with pathogenic or likely pathogenic variants on even a single allele were excluded, regardless of whether the gene followed an autosomal recessive pattern of inheritance. These cases were re-evaluated by the attending physician to identify any potential history, including past occurrences, that could have contributed to the condition.

**Fig. 1:**
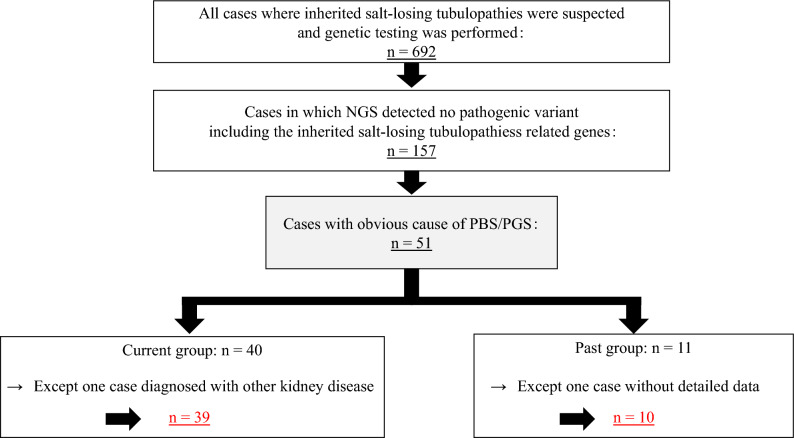
Flowchart of the methodology of selecting the research participants

Then we diagnosed PBS/PGS in 51 cases where a clear medical history of a causative factor of PBS/PGS (e.g., medication, complementary alternative medicines (CAM) and lifestyle history) could be confirmed. These patients were categorized into two groups: the current group (*n* = 40), in which the causative factors persisted at the time of examination, and the past group (*n* = 11), in which at least 1 year or more had elapsed since the elimination of the causative factors. One patient in the current group was excluded because he was diagnosed with idiopathic granulomatous interstitial nephritis based on renal biopsy results. In addition, one patient in the past group was excluded because of the unavailability of detailed laboratory data (Fig. 1). Based on the above, in this study, we conducted a retrospective comparative analysis of clinical characteristics and blood test results, including hypokalemia and kidney function, between the current group (*n* = 39) and the past group (*n* = 10).

The definition of excessive dieting was an intentional weight loss of 5% or more over a 6–12-month period. Emaciation was defined as a sustained body mass index (BMI) of less than 17 kg/m^2^, which corresponds to moderate anorexia nervosa in the Diagnostic and Statistical Manual of Mental Disorders, 5th edition [9]. In addition, according to data from the Ministry of Health, Labour and Welfare, the average BMI of Japanese women generally ranges from 20 to 23 and tends to be lower than that of women in other countries, and it is unclear how much weight loss is necessary to develop PBS/PGS.

### Genetic analysis

Comprehensive genetic analysis was performed in a stepwise manner. First, genomic DNA was extracted from peripheral blood leukocytes obtained from the patients and their family members using the Quick Gene Mini 80 system (Wako Pure Chemical Industries). Second, targeted exome sequencing by NGS using a custom disease panel for 136 genes related to inherited kidney diseases (Supplementary Table 1) was performed. NGS samples were prepared using a HaloPlex Target Enrichment System Kit (Agilent Technologies) in accordance with the manufacturer’s instructions. Amplified target libraries were sequenced using MiSeq (Illumina) and analyzed with SureCall (v.3.0; Agilent Technologies). Detected variants were confirmed by Sanger sequencing, and their pathogenicity was assessed using methods described later. In addition, for cases in which no pathogenic variants were detected by the above examinations, screening for CNVs in the SLT-related genes was conducted using pair analysis. All CNVs identified by pair analysis were confirmed by MLPA with SALSA (MRC Holland) or array CGH (Agilent Technologies), depending on the targeted genes.

### Variant evaluation

The pathogenicity of the detected variants was assessed according to the American College of Medical Genetics and Genomics (ACMG) and the Association for Molecular Pathology (AMP) guidelines as well as allele frequencies and pathogenicity determinations from genome databases such as the Genome Aggregation Database (gnomAD, https://gnomad.broadinstitute.org/), ClinVar (https://www.ncbi.nlm.nih.gov/clinvar/), and the Human Gene Mutation Database (HGMD, https://portal.biobase-international.com/hgmd/pro/start.php) [10]. Cases with variants classified as “Likely pathogenic” or “Pathogenic” according to the guideline or existing genome databases were excluded from the study. For variants classified as having “uncertain significance”, further investigations were conducted, referencing allele frequencies in the Japanese population based on the Japanese Multi-Omics Reference Panel (jMorp, https://jmorp.megabank.tohoku.ac.jp/202001/) and the Human Genetic Variation Database (HGVD, http://www.hgvd.genome.med.kyoto-u.ac.jp/)) and the results of segregation analysis in our cohort, and then cases with variants for which pathogenicity could not be definitively ruled out were excluded from the study.

### Statistical analyses

For statistical analysis, the Mann–Whitney U test was employed using GraphPad Prism 9.5.1 for Windows (GraphPad Software, Boston, Massachusetts USA, www.graphpad.com). All analyses were two-tailed, and the significance threshold was set at *p* < 0.05.

## Results

The characteristics and clinical manifestations of the patients in each group are shown in Table 1. The median age at the time of genetic testing was 44 years (range 16–60 years) in the current group and 38 years (range 29–58 years) in the past group. All but two patients in the current group were female. Body weight (median 39.0 vs. 51.2 kg *p* = 0.012) and BMI (median 16.7 vs. 19.4 kg/m^2^, *p* = 0.0031) were both significantly higher in the past group. In the past group, the median time since the elimination of causes was 7.5 years (range: 1–18 years). No obvious hypertension or hypotension was observed except for one case of hypertension in the current group. Diagnostic opportunities included numerous incidental blood tests conducted during routine checkups, screening for infectious diseases, and monitoring the progress of other diseases in both groups. Very few cases were diagnosed with the main complaint of symptoms related to hypokalemia, such as numbness, tetraplegia, tetany, muscle weakness, and fatigue (Table 1).

**Table 1:** Characteristics and clinical manifestations of the PBS/PGS^a^ syndrome cohort at the time of genetic analysis

	Current group (*n* = 39)	Past group (*n* = 10)	*p* value
Age (year, median)	44.0 (16–60)	37.5 (29–58)	0.4291
Sex (Male/Female)	2/37	0/10	–
Height (cm, median)	156.0 (147.0–171.0)	157.9 (149.0–165.4)	0.2205
Body weight (kg, median)	39.0 (22.3–87.3)	51.2 (37.7–64.0)	0.0116
Body mass index (kg/m^2^, median)	16.7 (9.6–35.4)	19.4 (17.0–26.0)	0.0031
Systolic blood pressure (mmHg, median)	100 (70–155)	101 (81–121)	0.9833
Diastolic blood pressure (mmHg, median)	61.5 (48–91)	67 (40–78)	0.8506
Elapsed period (years, median)	-	7.5 (1–18)	–
Diagnostic opportunity			
Blood test by chance^b^	32 (82.1%)	9 (90%)	–
PBS/PGS-related symptoms^c^	7 (17.9%)	1 (10%)	–

^a^PBS/PGS: Pseudo-Bartter/Gitelman syndrome

^b^Blood tests were conducted for routine checkups, screening for infectious diseases, and monitoring the progress of other medical conditions

^c^Symptoms include numbness and paralysis of the extremities, tetany, muscle weakness, and fatigue

In both groups, emaciation and excessive dieting emerged as the predominant factors contributing to PBS/PGS, accounting for 74.4% in the current group and 80% in the past group, followed by laxative abuse (Table 2). No CAM were confirmed, except for one case in the current group where the individual regularly consumed Chinese herbal tea. We identified several cases with underlying conditions such as anorexia nervosa, depression, panic disorder, and short bowel syndrome (SBS) as potential causes of emaciation. Specifically, two cases in the past group and four cases in the current group were diagnosed with anorexia nervosa. In addition, both the past and current groups had one case of depression each. One case in the past group had panic disorder, and one case in the current group had SBS following a subtotal colectomy for ulcerative colitis; however, in most cases, the cause remained unknown. In the current group, no treatment for these underlying conditions had been initiated at the time of genetic analysis, except for the SBS case. In the past group, there is no record of pharmacological treatment for the two cases diagnosed with anorexia nervosa, and the treatment details for cases with panic disorder and depression could not be confirmed.

**Table 2:** Causes of PBS/PGS^a,b^

	Current group	Past group
Emaciation^c^	28 (71.8%)	4(40%)
Excessive dieting	1 (2.6%)	4 (40%)
Laxatives^d^	15(38.5%)	2 (20%)
Alcoholism	3 (7.7%)	0
Diuretic (Furosemide)	2 (5.1%)	0
Chronic diarrhea^e^	2 (5.1%)	0
Habitual vomiting	1 (2.6%)	0
Habit of drinking Chinese tea	1 (2.6%)	0
Intoxication (Toluene)	0	1 (10%)

^a^PBS/PGS: Pseudo-Bartter/Gitelman syndrome

^b^The factors in the table may contain overlapping elements as they can merge

^c^Causes of emaciation include anorexia, depression, panic disorder, and short bowel syndrome

^d^Laxatives include magnesium oxide, sennoside, pantethine, bisacodyl, sodium picosulfate hydrate, and lubiprostone

^e^Identified causes of chronic diarrhea were irritable bowel syndrome and ulcerative colitis

Blood tests conducted before the initiation of treatment revealed evident hypokalemia in both the current and past groups, with a median of 2.4 vs. 2.6 mEq/L, respectively (*p* = 0.51). The median serum magnesium concentration in the current and past groups was 1.89 mg/dL vs. 1.85 mg/dL, respectively (*p* = 0.99) (Table 3). No significant differences were observed between the two groups in terms of electrolyte parameters or venous blood gas results. Notably, overactivation of the renin–angiotensin system was observed in both groups, as evidenced by a median plasma renin activity (PRA) of 21.2 vs. 13.2 ng/mL/h (*p* = 0.24) and a median plasma aldosterone concentration (PAC) of 253 vs. 165 pg/mL (*p* = 0.031), in the current and past groups, respectively. While the past group showed a statistically significant decrease in PAC, this finding may imply that causative factor elimination was accomplished. Renal dysfunction was observed in both groups, with no significant differences in renal function at the time of genetic testing. The median serum creatinine level (Cr) was 0.86 vs. 0.83 mg/dL (*p* = 0.91) and the median estimated glomerular filtration rate (Cr-eGFR) [11] was 58 vs. 61 mL/min/1.73 m^2^ (*p* = 0.84) in the current and past groups, respectively (Table 3).

**Table 3:** Blood test results before initiation of treatments and at the time of genetic testing

	Current group (*n* = 39)	Past group (*n* = 10)	*p* value
Before initiationof treatments			
K (mEq/L, median)	2.4 (1.5–3.4)	2.55 (1.7–2.9)	0.5116
Na (mEq/L, median)	138 (130–149)	139 (136–141)	0.7225
Cl (mEq/L, median)	91 (78–105)	96 (89–106)	0.2087
Mg (mg/dL, median)	1.89 (0.7–3.3)	1.85 (0.8–2.3)	0.985
pH (median)	7.474 (7.337–7.573)	7.452 (7.342–7.574)	0.4188
Base excess (mEq/L, median)	9.15 (⁻7.1–23.0)	8.8 (2.8–27.1)	0.6521
HCO_3_^−^ (mEq/L, median)	33.35 (17.0–49.9)	33.1 (24.1–56.3)	0.9339
PRA^a^ (ng/mL/hr, median)	21.2 (3.9–91.4)	13.2 (0.8–20.0)	0.2393
PAC^b^ (pg/mL, median)	253 (17.7–3680)	165 (36–251)	0.031
At the time of genetic testing			
BUN (mg/dL, median)	15.0 (4.4–45.0)	12.1 (4.0–38.2)	0.5051
Cr (mg/dL, median)	0.855 (0.41–3.10)	0.83 (0.48–1.34)	0.9113
Cr-eGFR (mL/min/1.73m^2^, median)	57.61 (16.1–119.2)	60.72 (34.3–118.4)	0.8442

^a^PRA: plasma renin activity

^b^PAC: plasma aldosterone concentration

Furthermore, we conducted another comparative analysis restricted to cases where excessive dieting or emaciation was identified as the sole cause of PBS/PGS. These cases amounted to 15 cases in the current group and 8 cases in the past group. In the past group, the cases were objectively confirmed to have complete removal of the causative agent at the time of genetic testing. As a result, the analysis findings for each item were consistent with those observed for the entire cohort as described above, except that there was no significant difference in PAC (Supplementary Table 2).

## Discussion

To the best of our knowledge, this is the first report indicating the persistence of PBS/PGS findings long after the elimination of the underlying cause. Clinically, it is noteworthy that even in cases where the causative factor has been resolved for over a year, patients continue to manifest the same degree of hypokalemia and renal dysfunction. Incidental blood tests are the most common diagnostic opportunity, suggesting that renal dysfunction may progress without awareness. In the past group, the causes were revealed in some cases for the first time when patients were re-interviewed after negative genetic test results for SLTs were reported. These observations emphasize the importance of detailed medical interviews and the necessity for an extended follow-up period in such cases.

Regarding pathogenesis, we suspect that prolonged hypokalemia may result in a low set point for serum potassium levels being fixed in the renal tubules. The specifics remain unclear, as there are currently no reports demonstrating this inference in vivo, in vitro, or describing cases analogous to those presented in this study, as far as we are aware. However, the observed persistent overactivation of the renin–angiotensin system after eliminating the underlying cause in the past group, similar to that in the current group, supports this hypothesis, albeit indirectly. No patients in the past group underwent longitudinal evaluation of the renin–angiotensin system, but one case in the current group (Bartter8 in Supplementary Table 3) did. She developed PBS/PGS due to prolonged laxative abuse, and despite the correction of the laxative dosage to an appropriate level, persistent activation of the renin–angiotensin system was confirmed [7]. To further elucidate the pathophysiology, a prospective long-term study is necessary to evaluate the correlation between the activation of the renin–angiotensin system and serum potassium levels in cases diagnosed as PBS/PGS.

Chronic hypokalemia has been reported as a factor causing renal impairment, termed hypokalemic nephropathy. The pathogenesis of this condition is mediated by an imbalance in vasoactive mediators, which leads to vasoconstriction and medullary ischemia. This process results in tubulointerstitial damage characterized by renal hypertrophy and renal tubular cell hyperplasia involving the medullary collecting ducts and, to a lesser extent, the thick ascending limb in association with tubular atrophy, interstitial macrophage infiltration, and interstitial fibrosis [12–14]. The reduction in urinary concentrating ability caused by hypokalemia is primarily attributed to the downregulation of aquaporin channels; however, the hyperactivation of the renin–angiotensin system, leading to increased sodium reabsorption in the proximal tubules, is also suspected to be involved [14]. Vacuolar degeneration of the proximal tubular epithelial cells is a well-known histological feature. However, to the best of our knowledge, no previous studies have reported the pathological findings of PBS/PGS cases such as those included in the present study. In the past group of this study, renal biopsy was performed in only one case (B027 in Supplementary Table 4). Nonetheless, immunofluorescence and electron microscopy findings were not available, and the details of the light microscopy findings were unclear. It was only reported that no hyperplasia of the juxtaglomerular apparatus was observed. To validate the hypothesis mentioned above, it is necessary to periodically examine PRA and PAC and to accumulate renal pathological findings in PBS/PGS cases.

This study has some limitations. First, as a retrospective study, it includes incomplete data for some parameters and details on the treatment of underlying conditions and hypokalemia. In particular, urinalysis results could not be confirmed in most cases, and potassium and calcium excretion levels could not be reviewed. As for biomarkers suggestive of tubular injury, including urinary β2-microglobulin, the number of cases for which test results were available was extremely limited, and therefore these markers were not included in the data collected for this study. Moreover, crucial factors for evaluating the renin–angiotensin system, such as the time of specimen collection, body position, body fluid volume, and the time course of recovery from emaciation, were also unclear due to insufficient data. Regarding the treatment of psychiatric disorders, the specific therapies used in the past group at that time are unknown, and it was not possible to determine whether medications such as quetiapine or lithium preparations, which can induce hypokalemia, were administered. Second, diseases other than PBS/PGS may have been present. We excluded other congenital renal tubular diseases by NGS analysis, such as autosomal dominant hypocalcemia, *HNF1B*-related nephropathy, cystic fibrosis, congenital chloride diarrhea, nephronophthisis, Dent’s disease, mitochondrial disease, renal tubular acidosis and congenital anomalies of the kidney and urinary tract, which are known to present with hypokalemia [2]. However, hypokalemia due to unidentified genetic abnormalities may have been present. Recently, it was reported that some pathogenic mitochondrial DNA (mtDNA) variants in the genes encoding the transfer RNAs for phenylalanine and isoleucine can cause a Gitelman-like syndrome and progressive renal dysfunction [15]. Although we were not able to search for these mtDNA variants in the present study, it is necessary to include the investigation for these variants in comprehensive genetic testing in the future, especially in cases with a maternal inheritance pattern. Third, the complete elimination of causes in the past group cases is crucial; however, this relies on the repeated interviews conducted by the attending physicians. There remains the possibility that patients may have continued using over-the-counter medications, such as laxatives, without disclosure. Lastly, we could not determine whether the severity of hypokalemia or the duration of exposure to the underlying cause of PBS/PGS influenced the persistence of hypokalemia after the cause was eliminated. As previously noted, although this study did not establish a strict correlation with past episodes and had some data limitations, our primary objective was to report the existence of such cases. To clarify these issues, it is necessary to accumulate cases with long-term follow-up after PGS/PGS diagnosis.

In conclusion, this study indicates that PBS/PGS findings, including hypokalemia, persist over an extended period, even after the removal of causative factors, indicating a potential link to the development of renal dysfunction. Furthermore, sustained activation of the renin–angiotensin system may be involved in this process. Given that persistent hypokalemia due to PBS/PGS can lead to renal dysfunction and even ESKD in some patients, this prolonged manifestation is clinically significant, emphasizing the need for prompt elimination of the cause and long-term follow-up to improve renal prognosis. However, the lack of previous reports on such cases leaves uncertainty around numerous aspects. Therefore, it is imperative to systematically accumulate additional cases in the future to gain a comprehensive understanding of the intricate pathophysiological mechanisms hypothesized above.

## Supplementary Information

Below is the link to the electronic supplementary material.

Supplementary file1 (PDF 343 KB)

## Data Availability

We have provided all data in the text and supplementary data file. The data underlying this article will be shared upon reasonable request by the corresponding authors.
