# Supplementary material for: Prolonged hypokalemia long after causative factor elimination in pseudo-Bartter/Gitelman syndrome
Source: Clin Exp Nephrol. 2025 Jul 25;29(12):1796–802. doi: 10.1007/s10157-025-02734-4 (PMC12660418; doi:10.1007/s10157-025-02734-4)
Supplement: Supplementary file 1 — Supplementary file1 (PDF 343 KB) [file 10157_2025_2734_MOESM1_ESM.pdf]

Supplementary Materials

Title:

Prolonged Hypokalemia Long After Causative Factor Elimination in Pseudo-Bartter/Gitelman Syndrome

Authors:

Atsushi Kondo, MD, PhD[1]*, Tomoko Horinouchi, MD, PhD[1], Yuta Inoki, MD[1],

Yuta Ichikawa, MD[1], Yu Tanaka, MD[1], Hideaki Kitakado, MD[1], Chika Ueda, MD[1],

Nana Sakakibara, MD, PhD[1], China Nagano, MD, PhD[1], Kandai Nozu, MD, PhD[1]

Lists:

Supplemental Table 1. The target gene list of next-generation sequencer

Supplemental Table 2. Blood test results before treatment initiation and at the time of genetic testing; limited to cases caused by emaciation or excessive dieting

Supplemental Tabe 3. Summary of cases in the current group

Supplemental Table 4. Summary of cases in the past group

Supplemental Table 1. The target gene list of next-generation sequencer

SLC12A1 NM_000338.2	CFI NM_000204.3	SCNN1G NM_001039.3	LAMB2 NM_002292.3	PTPRO NM_030667.2	LAGE3 NM_006014.4
KCNJ1 NM_000220.4	MCP (CD46) NM_002389.4	KLHL3 NM_017415.2	SCARB2 NM_005506.3	XPO5 NM_020750.2	OSGEP NM_017807.3
CLCNKB NM_000085.4	C3 NM_000064.3	CUL3 NM_003590.4	COQ2 NM_015697.7	ACTN4 NM_004924.4	TPRKB NM_016058.3
BSND NM_057176.2	CFB NM_001710.5	WNK1 NM_018979.3	COQ6 NM_182476.2	ANLN NM_018685.4	TP53RK NM_033550.3
CLCNKA NM_004070.3	DGKE NM_003647.2	WNK4 NM_032387.4	ITGA3 NM_002204.2	ARHGAP24 NM_001025616.2	WDR4 NM_18669.5
SLC12A3 NM_000339.2	THBD NM_000361.2	AQP2 NM_000486.5	ITGB4 NM_001005731.1	INF2 NM_022489.3	WDR73 NM_032856.3
CASR NM_000388.3	CFHR1 NM_002113.2	AVPR2 NM_000054.4	GLEPP1 (PTPRO) NM_030667.2	LMX1B NM_002316.3	PRDM15 NM_001040424.3
MAGED2 NM_177433.2	ADAMTS13 NM_139025.4	AVP NM_000490.4	MYO1E NM_004998.3	MYH9 NM_002473.5	TRIM8 NM_030912.2
CLDN10 NM_006984.4	FN1 NM_212482.1	COL4A3 NM_000091.4	ARHGDIA NM_001185077.2	PAX2 NM_003987.3	PODXL NM_005397.3
CFTR NM_000492.3	SLC4A1 NM_000342.3	COL4A4 NM_000092.4	ADCK4 NM_024876.3	TRPC6 NM_004621.5	TBC1D8B NM_017752.2
CLCN5 NM_000084.4	ATP6V0A4 NM_020632.2	COL4A5 NM_000495.4	TTC21B NM_024753.4	WT1 NM_24426.5	MAFB NM_005461.5
OCRL NM_000276.3	ATP6V1B1 NM_001692.3	P3H2 NM_018192.4	NUP93 NM_014669.4	MAGI2 NM_012301.3	
SLC26A3 NM_000111.2	SLC4A4 NM_003759.3	CD151 NM_004357.5	NUP107 NM_020401.2	AVIL NM_ 006576.3	
KCNJ10 NM_002241.4	CA2 NM_000067.2	GLA NM_000169.2	NUP205 NM_015135.2	TNS2 NM_015319.2	
CLDN16 NM_006580.3	EHHADH NM_001966.3	UMOD NM_003361.3	NUP85 NM_024844.4	DLC1 NM_182643.2	
CLDN19 NM_148960.2	SLC34A1 NM_003052.4	MUC1 NM_002456.5	NUP133 NM_018230.2	CDK20 NM_001039803.2	
FXYD2 NM_001680.4	SLC2A2 NM_000340.1	SEC61A1 NM_013336.3	NUP160 NM_015231.1	ITSN1 NM_001001132.1	
EGF NM_001963.4	BCS1L NM_001257343	REN NM_000537.3	CRB2 NM_173689.6	ITSN2 NM_147152.2	
TRPM6 NM_017662.4	GATM NM_001482.2	EYA1 NM_000503.5	CUBN NM_001081.3	KIRREL1 NM_018240.6	
KCNA1 NM_000217.2	HNF4A NM_175914.4	SIX2 NM_016932.4	EMP2 NM_001424.4	SGPL1 NM_003901.3	
CNNM2 NM_017649.4	NDUFAF6 NM_152416.3	CD2AP NM_012120.2	FAT1 NM_005245.3	LMNA NM_170707.3	
HNF1B NM_000458.3	CTNS NM_004937.2	NPHS1 NM_004646.3	KANK1 NM_015158.3	LAMA5 NM_005560.4	
PCBD1 NM_000281.3	NR3C2 NM_000901.4	NPHS2 NM_014625.3	KANK2 NM_001136191.2	GAPVD1 NM_ 015635.3.	
ANK3 NM_020987.5	SCNN1A NM_001038.5	PLCE1（NPHS3）NM_016341.3	KANK4 NM_181712.4	ANKFY1 NM_ 001257999.2.	
CFH NM_000186.3	SCNN1B NM_000336.2	SMARCAL1 NM_014140.3	PDSS2 NM_020381.3	GON7 NM_032490.4	

Supplemental Table 2. Blood test results before treatment initiation and at the time of genetic testing; limited to cases caused by emaciation or excessive dieting

		Current group (n=15)	Past group (n=8)	p value
Before initiation of treatments	K (mEq/L, median)	2.3 (1.8-3.3)	2.5 (1.7-2.9)	0.2053
	Na (mEq/L, median)	137.0 (130-147)	138.5 (136-141)	0.3879
	Cl (mEq/L, median)	90 (78-94)	92 (89-100)	0.25
	Mg (mg/dL, median)	1.90 (0.9-2.5)	1.85 (0.8-2.2)	0.7633
	pH (median)	7.490 (7.350-7.540)	7.472 (7.454-7.574)	0.6011
	Base excess (mEq/L, median)	10.45 (-3.0-23.0)	11.10 (2.8-27.1)	0.9044
	HCO3- (mEq/L, median)	33.6 (21.9-49.9)	35.9 (31.3-56.3)	0.5121
	PRA[1)] (ng/mL/hr, median)	41.55 (7.8-91.4)	18.20 (6.7-36.0)	0.0533
	PAC[2)] (pg/mL, median)	215 (102-2090)	205 (104-251)	0.4069
At the time of genetic testing	BUN (mg/dL, median)	16.6 (8.6-42.0)	12.1 (4.0-38.2)	0.3837
	Cr (mg/dL, median)	0.93 (0.59-3.10)	0.83 (0.50-1.34)	0.8736
	Cr-eGFR (mL/min/1.73m2, median)	53.35 (16.15-92.42)	60.72 (34.27-113.19)	0.8044

1) PRA: plasma renin activity

2) PAC: plasma aldosterone concentration

Supplemental Tabe 3. Summary of cases in the current group

ID	Age	Sex[1)]	HT (cm)	BW (kg)	BMI (kg/m^2)	sBP (mmHg)	dBP (mmHg)	Cause	Diagnostic opportunity	Blood tests[2)]											
										before initiation of treatments									at the time of genetic testing		
										Serum				Plasma		Venous blood gas			Serum		
										K (mEq/L)	Na (mEq/L)	Cl (mEq/L)	Mg (mg/dL)	PRA (ng/mL/hr)	PAC (pg/mL)	pH	BE (mEq/L)	$HCO3^-$ (mEq/L)	UN (mg/dL)	Cr (mg/dL)	Cr-eGFR ($mL/min/1.73m^2$)
B184	36	F	152.8	39	16.7	115	80	Emaciation	PBS/PGS-related symptoms[4)]	2.6	ND	ND	0.9	7.8	131	7.54	9.3	32.5	15	0.67	79.44
B189	20	F	160	37	14.5	91	51	Emaciation	Blood test by chance[3)]	1.9	ND	ND	2.5	19.3	214.7	7.52	21.8	44.6	8.6	0.83	74.40
B194	37	F	151	34	14.9	ND	ND	Emaciation<br>Laxatives	Blood test by chance	2.9	ND	ND	2.1	3.9	120	7.486	8.2	32.2	11.1	0.73	71.76
B202	60	F	157.3	65.6	26.5	113	67	Diuretics	PBS/PGS-related symptoms	3.3	ND	ND	0.7	37	857	7.408	3	27.8	ND	ND	ND
B216	60	F	160	44	17.2	100	60	Emaciation<br>Laxatives	Blood test by chance	2.5	ND	ND	1.7	9.4	165	7.427	14.8	40	10	0.78	58.10
B233	27	F	155	35.9	14.9	75	55	Emaciation	Blood test by chance	2.3	ND	ND	1.9	41.1	281.4	7.51	16	41	42	3.1	16.15
B236	57	F	162.1	44.3	16.9	98	50	Emaciation	Blood test by chance	2.6	ND	ND	1.7	70.9	2090	7.476	5.8	29.4	20.7	0.93	48.64
B268	40	F	159.4	41.4	16.3	87	54	Emaciation	PBS/PGS-related symptoms	1.8	ND	ND	1.7	70.6	309	7.437	7.7	33.1	ND	ND	ND
B274	45	F	159.4	43.9	17.3	103	67	Emaciation<br>Laxatives	Blood test by chance	2	ND	ND	2.4	8	280	7.45	6	31.5	11	0.51	100.44
B278	44	F	157	87.3	35.4	155	85	Chronic diarrhea	Blood test by chance	2.9	ND	ND	0.8	20	457	7.47	15	40	11.9	0.68	73.79
B280	34	F	152	38	16.4	96	59	Emaciation	Blood test by chance	2.45	ND	ND	2	70	688	7.501	14.5	40	21.4	1.07	48.39
B293	50	F	154	46	19.4	113	81	Chronic diarrhea	Blood test by chance	2.6	ND	ND	1.4	55.3	311	7.491	6.4	30.2	17.7	0.91	51.72
B294	54	F	167	45	16.1	ND	ND	Emaciation	Blood test by chance	2.1	ND	ND	1.6	27.7	186	ND	ND	ND	13.8	0.84	55.22
B295	52	M	171	74.1	25.3	109	81	Habit of drinking Chinese tea	PBS/PGS-related symptoms	2.2	ND	ND	1.8	22.8	189	7.503	11.9	36.5	22.9	1.19	38.13
B302	36	F	157.3	41.3	16.7	95	64	Emaciation<br>Laxatives	Blood test by chance	2.3	ND	ND	ND	57.7	617	7.449	12	37	13	0.6	89.64
B307	30	F	149.5	32.9	14.7	113	78	Emaciation<br>Laxatives	Blood test by chance	2.8	ND	ND	3.3	13	3680	7.53	13	37.6	45	2.79	17.58
B316	50	F	155	29.5	12.3	78	56	Emaciation	PBS/PGS-related symptoms	2.6	ND	ND	2	8.5	215	7.527	11.6	33	18	0.61	80.11
B327	29	F	157	47.6	19.3	105	75	Laxatives	Blood test by chance	2.4	ND	ND	2.2	37.8	625	7.405	-0.5	23.5	10.9	0.53	109.24
B349	45	F	147.8	47.3	21.7	89	54	Laxatives	Blood test by chance	3.4	ND	ND	2.1	9.6	827	7.42	7.8	33.5	15.1	1.24	38.00
B361	57	F	148.7	32.4	14.7	116	72	Emaciation<br>Laxatives	Blood test by chance	2.3	ND	ND	1.6	6.9	253	7.428	5.1	29.7	23.1	1.47	29.48
B363	63	F	151.3	33.4	14.6	102	61	Emaciation<br>Alcoholism	Blood test by chance	3.1	ND	ND	1.88	6.7	233.7	7.408	13.9	41.9	36.5	0.97	45.13
B378	27	F	164.3	53.6	19.9	98	65	Laxatives	Blood test by chance	2.5	ND	ND	1.6	26.7	ND	7.459	11.9	37.9	12.1	0.87	64.84
B387	16	F	156	46.2	19.0	86	54	Habitual vomiting	Blood test by chance	3.2	142	97	1.6	7.1	117	7.5	11.3	35.9	18.4	0.89	73.49
B395	26	F	151	38.1	16.7	102	60	Emaciation	Blood test by chance	3.3	137	90	1.4	42	2140	7.46	8	33.2	9.2	1.05	53.35
B409	30	F	147	37	17.1	111	79	Emaciation<br>Laxatives	Blood test by chance	2.2	ND	ND	2.2	90.1	1671.7	7.486	10.6	35	19.3	0.53	108.18
B441	36	F	160.8	47.6	18.4	104	71	Laxateives	Blood test by chance	3	138	94	2	7.7	194	7.472	7.6	32.3	7.5	0.7	75.73
B444	56	F	147	36	16.7	100	67	Emaciation<br>Laxateives	Blood test by chance	1.8	144	88	2.9	27.2	125.7	7.498	8.1	31.8	15	1.18	37.68
B462	26	F	150	38	16.9	ND	ND	Emaciation	Blood test by chance	2.3	137	87	2.3	91.4	292	7.467	16.5	43.8	ND	ND	ND
B465	40	F	156.8	40	16.3	98	58	Emaciation<br>Diuretics	PBS/PGS-related symptoms	1.6	133	85	1.9	4	17.7	7.51	7	30.3	16	0.94	53.22
B480	57	M	160	53.56	20.9	127	88	Alcoholism<br>Excessive dieting	Blood test by chance	1.5	139	91	1.5	ND	ND	7.573	16.6	41.5	4.4	0.41	119.16
B481	66	F	156	30	12.3	87	50	Emaciation	Blood test by chance	1.9	130	94	1.6	61.8	194	7.47	6	30	27.7	2.22	18.00
B488	48	F	152	37	16.0	110	58	Emaciation<br>Laxatives	Blood test by chance	2.5	138	105	1.8	6.8	253	7.421	0.3	24.7	13	0.84	57.12
B489	57	F	153	31.4	13.4	93	62	Emaciation<br>Laxatives	Blood test by chance	2.9	139	96	1.9	4.7	204	7.35	-7.1	17	6.6	0.67	69.63
B495	56	F	157	63	25.6	136	91	Alcoholism	Blood test by chance	1.8	149	94	1.1	ND	ND	7.514	11.1	35.2	6.4	0.57	83.52
B503	57	F	161	37	14.3	98	61	Emaciation	PBS/PGS-related symptoms	2.1	130	90	2.4	63.2	102.1	7.479	9	34	16.2	0.59	80.02
B516	42	F	163	32.6	12.3	85	48	Emaciation	Blood test by chance	2.3	139	80	1.9	10.5	102	7.519	22.3	48.6	9.8	1.06	46.01
B540	19	F	152.8	22.3	9.6	70	53	Emaciation	Blood test by chance	1.8	136	78	1.2	21.2	108	7.51	23	49.9	ND	0.69	92.42
B541	53	F	162	41	15.6	92	54	Emaciation	Blood test by chance	2.3	147	91	2.3	ND	425.4	7.35	-3	21.9	17	1.05	43.49
Bartter8	33	F	150	35.3	15.7	106	68	Emaciation<br>Laxatives	Blood test by chance	2.8	ND	ND	2.7	ND	1260	7.337	-2.9	23	22	1.63	30.80

1) F: female, M: male

2) ND: no data

3) Blood tests were conducted for routine check-ups, screening for infectious diseases, or monitoring the progress of other medical conditions.

4) The symptoms include numbness and paralysis of the extremities, tetany, muscle weakness, and fatigue.

5) Characteristics of the patients such as age, HT, BW, BMI, and BP are at the time of genetic analysis.

Supplemental Table 4. Summary of cases in the past group

ID	Age	Sex[1]	HT (cm)	BW (kg)	BMI (kg/m^2)	sBP (mmHg)	dBP (mmHg)	Cause	Diagnostic opportunity	Elapsed Period (years)	Blood tests[2]											
											before initiation of treatments									at the time of genetic testing		
											Serum				Plasma		Venous blood gas			Serum		
											K (mEq/L)	Na (mEq/L)	Cl (mEq/L)	Mg (mg/dL)	PRA (ng/mL/hr)	PAC (pg/mL)	pH	BE (mEq/L)	$HCO3^-$ (mEq/L)	UN (mg/dL)	Cr (mg/dL)	Cr-eGFR (mL/min/1.73m^2)
B027	29	F	159	44	17.4	ND	ND	Excessive dieting (BW loss of 30 kg within one year at age 17)	Blood test by chance[3]	12	2.9	ND	ND	1.6	18.2	251	7.454	21.6	46.6	12.7	1.2	44.68
B180	39	F	149	37.7	17.0	109	70	Emaciation	Blood test by chance	2	2.5	ND	ND	1.6	13.2	205	7.51	8.8	31.3	10.4	1.22	40.30
B296	49	F	160.5	49.4	19.2	101	72	Laxatives	Blood test by chance	1	2.6	ND	ND	1.8	0.8	37.5	7.342	ND	24.1	19	1.13	41.05
B373	32	F	157.3	44	17.8	81	51	Ematiation	Blood test by chance	14	1.7	ND	ND	0.8	ND	ND	7.574	27.1	56.3	4	0.5	113.19
B375	58	F	165.4	53.6	19.6	121	65	Excessive dieting (BW loss of 50 kg within 1.5 years at age 40)	Blood test by chance	18	2.7	136	ND	2.1	11.5	163	ND	ND	ND	11.5	0.7	66.04
B386	23	F	156	55	22.6	102	68	Excessive dieting, Laxatives (Regular BW loss of 10 kg within one month 1-2 times per year over 4 years, from ages 17 to 21)	Blood test by chance	2	2.2	ND	ND	1.8	20	165	7.49	13.4	35.9	13	0.67	90.35
B392	36	F	158.5	43.8	17.4	100	67	Emaciation (BMI 8.8 at age 33)	PBS/PGS-related symptoms[4]	3	2.5	139	89	2	36	247	ND	ND	ND	11.4	0.8	65.43
B396	32	F	156	53	21.8	90	40	Intoxication (toluene)	Blood test by chance	10	2.8	141	106	2.3	10.9	36	7.403	3.4	28.3	9.2	0.48	118.36
B418	48	F	163	53	19.9	85	52	Emaciation (BMI 13.9 at age 43)	Blood test by chance	5	2.4	138	92	2.2	20	104	7.4	8.3	33.1	38.2	1.34	34.27
B501	47	F	157	64	26.0	118	78	Excessive dieting (BW loss of 30kg within one year at age 30)	Blood test by chance	17	2.8	141	100	1.9	6.7	235	7.45	2.8	ND	22.9	0.86	56.00

1) F: female, M: male

2) ND: no data

3) Blood tests were conducted for routine check-ups, screening for infectious diseases, or monitoring the progress of other medical conditions.

4) The symptoms include numbness and paralysis of the extremities, tetany, muscle weakness, and fatigue.

5) Characteristics of the patients such as age, HT, BW, BMI, BP, and elapsed period are at the time of genetic analysis.
